# Topical Treatment of Actinic Keratosis and Metalloproteinase Expression: A Clinico-Pathological Retrospective Study

**DOI:** 10.3390/ijms231911351

**Published:** 2022-09-26

**Authors:** Elena Campione, Monia Di Prete, Cosimo Di Raimondo, Gaetana Costanza, Vincenzo Palumbo, Virginia Garofalo, Sara Mazzilli, Chiara Franceschini, Emi Dika, Luca Bianchi, Augusto Orlandi

**Affiliations:** 1 Dermatology Unit, Department of Systems Medicine, University of Rome Tor Vergata, 00133 Rome, Italy; 2 Anatomic Pathology Unit, Department of Biomedicine and Prevention, University of Rome Tor Vergata, 00133 Rome, Italy; 3 Division of Dermatology, Azienda Ospedaliero-Universitaria di Bologna, 40126 Bologna, Italy; 4 Division of Dermatology, Department of Experimental Diagnostic and Specialty Medicine (DIMES), University of Bologna, 40126 Bologna, Italy

**Keywords:** actinic keratosis, immunohistochemistry, ingenol mebutate, metalloproteinases, piroxicam, photodynamic therapy, topical therapy

## Abstract

Actinic keratosis is an intraepithelial proliferation of atypical keratinocytes that could progress into invasive squamous cell carcinoma. Most evidence suggests an important role of the dermal matrix metalloproteinases in the progression of atypical skin epithelial lesions. We evaluated the clinical efficacy of three different therapeutic modalities (a medical device containing 0.8% piroxicam cream and 50+ sunscreen, photodynamic therapy, and ingenol mebutate gel) to treat suspicious actinic keratoses, which were biopsied for histopathological examination and then analyzed for the expression of matrix metalloproteinases by immunohistochemistry. Clinical, dermoscopic, and reflectance confocal microscopy evaluations revealed a gradual decrease in all standard scores validated for actinic keratosis assessment at the end of the treatments. From a histopathological point of view, we documented the substantial restoration of normal skin architecture, while the immunohistochemical evaluation of matrix metalloproteinases showed a reduction in expression in the treated skin lesions compared to the baseline. As actinic keratoses are considered the precursors of squamous cell carcinoma, their treatment is crucial to prevent the development of a more aggressive disease. Our study monitored the evolution of actinic keratoses subjected to three different topical therapies, with the value of correlating clinical and histopathological findings. Moreover, as the matrix metalloproteinases are largely recognized factors involved in the pathogenesis and evolution of actinic keratosis to squamous cell carcinoma, the demonstration by immunohistochemistry of a reduction in their expression after the treatments adds new valuable concern to the field.

## 1. Introduction

Actinic keratosis (AK) is an intraepithelial proliferation of atypical keratinocytes that could evolve to invasive squamous cell carcinoma (SCC). Many Authors consider AK to be an in situ SCC that may progress to the invasive stage [1,2]. Ultraviolet (UV) radiation is the principal cause of non-melanoma skin cancer (NMSCs) development, including AK, SCC, Bowen’s disease, and basal cell carcinoma (BCC) [3]. AKs usually arise in fair skin, mainly in elderly patients with a history of chronic sun exposure [4]. The rates of progression to invasive SCC have been estimated at 0–0.075% per AK per year for individuals without a previous history of NMSCs and 0–5% for those with a prior history of NMSCs [5,6]. Generally, AKs are slowly growing tumors. Thus, diagnosis in early stages permits a conservative approach and a higher cure rate for the disease [7]. Nowadays, clinicians apply useful non-invasive imaging techniques such as dermoscopy and reflectance confocal microscopy (RCM), which are helpful not only in diagnosing but also in monitoring the effect of treatments over time [8,9,10]. Clinically regressed lesions may show features that are still indicative of AKs through dermoscopy or RCM. Most evidence suggests a crucial role of the dermal matrix metalloproteinases (MMPs) in the progression of atypical skin epithelial lesions [11]. Due to their extensive extracellular matrix (ECM)-remodeling property, MMP activity is involved in several normal and pathologic processes, including tumor growth, progression, and metastasis [12]. The degradation of ECM components, such as collagen, fibronectin, elastin, and proteoglycans, by MMPs is responsible for premature aging of the skin [13]. UV radiation increases human skin MMP expression [14,15]. Five families of MMPs are currently recognized, but MMP-1 and 2 seem to favor the evolution of AKs [16]. MMP-1 degrades fibrillar collagen types I and III into specific fragments at a single site within the central triple helix as an initial step in tumor growth [13,17]. MMP-2 shows proteolytic activity against components of the basement membrane and plays a key role in the invasiveness and metastasis of NMSCs [13,18]. Thus, these proteases have become crucial therapeutic targets for the treatment and prevention of AKs and SCCs [19,20]. There is no gold standard for the therapy of AKs [21,22], but dermatologists refer to guidelines for choosing the best treatment modality considering a lesion’s features and the extension of the field of cancerization (FC) [23,24,25]. Therapies for AKs are currently classified according to their pathogenetic target. Anti-inflammatory molecules, i.e., 3% diclofenac and a medical device containing 0.8% piroxicam, inhibit the activity of cyclooxygenases isoenzymes 1 and 2 and, consequently, angiogenesis and induce apoptosis in dysplastic keratinocytes. By applying a topical photosensitizing substance, photodynamic therapy (PDT) induces reactive oxygen species (ROS) production, thus resulting into cell death, either by necrosis or apoptosis. Immunomodulators, i.e., imiquimod and ingenol mebutate (IM), stimulate both innate and adaptive cell-mediated immune responses. Retinoids have antiproliferative properties and favor keratinocyte differentiation [23]. In our study, we documented the effect of three different AK therapeutic modalities [26,27,28]: (1) a medical device containing 0.8% piroxicam cream and 50+ sunscreen (hereafter designated only piroxicam), (2) PDT, and 3) 0.015% or 0.05% IM gel (which EMA suspended from marketing at the end of 2019) [29]. For each patient, we evaluated through dermoscopy, RCM, and a histopathological examination the suspicious AK features before and after the treatments. In addition, we analyzed, by immunohistochemistry, the expression of MMP-1 and MMP-2 in the epithelial and stromal components in the histopathological specimens.

## 2. Results

### 2.1. Dermoscopic and RCM Evaluations

**Figure 1:**
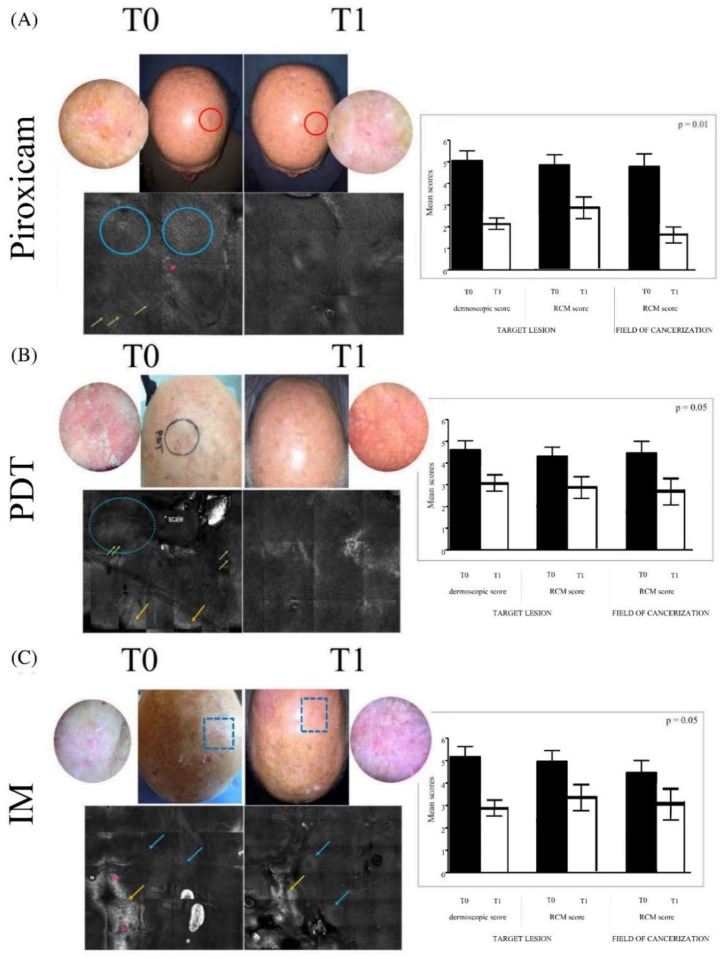
Clinical, dermoscopic, and reflectance confocal microscopy evaluation before (T0) and after treatment (T1). In the panels on the left, the images represent the clinical aspects of the target lesion and the field of cancerization of the scalps of different patients treated with different therapies: (**A**) medical device 0.8% piroxicam and 50+ sunscreen, (**B**) photodynamic therapy (PDT), (**C**) 0.015% ingenol mebutate (IM) gel; the dermoscopic features of the target lesion, in detail; and reflectance confocal microscopy mosaic (6 × 6) aspects of the target lesion and the field of cancerization. The blue circles and arrows indicate the atypical honeycombing pattern, constituted by pleomorphic keratinocytes; the red asterisks areas of detached keratinocytes; the green arrows inflammatory infiltrate; and the yellow arrows hyper- and para-keratosis. In the panels on the right, we report the dermoscopic and reflectance confocal microscopy scores of the target lesion and the reflectance confocal microscopy scores of the field of cancerization for the respective treatments.

Thirty patients were included in this retrospective study, and they were divided into three homogeneous groups that were treated with different regimens. All patients’ characteristics are reported in Table 1. The evaluation of clinical, dermoscopic, and RCM parameters revealed substantial differences between the first and last visit in all three groups (Figure 1). Patients treated with the medical device piroxicam (group A) were examined after 4 weeks of treatment and then re-evaluated at 16 weeks from baseline. A reduction was recorded in the mean value of the scores in dermoscopy and RCM regarding the target lesions from 5 to 2.1 and from 4.8 to 2.9 respectively, while the mean score in RCM went from 4.9 to 1.7 for the FC (Figure 1A, right; *p* = 0.01). Patients treated with PDT (group B) received a total of two cycles of treatment with a break of 4 weeks in between and were re-evaluated at 16 weeks from baseline. The dermoscopic mean score of the target lesion highlighted a reduction from 4.7 to 3.1. The RCM mean scores detected reductions from 4.4 to 3.0 and from 4.6 to 2.9, respectively, for the target lesion and the FC (Figure 1B, right; *p* = 0.05). Patients treated with IM (group C) received a single cycle of treatment for three days and were re-evaluated at 16 weeks from baseline. We detected a reduction in the mean value of the dermoscopic score from 5.1 to 2.9 in the target lesion. For the RCM score, there was a reduction from 4.8 to 3.4 and from 4.1 to 3.1 for the target lesion and the FC, respectively (Figure 1C, right; *p* = 0.05). In all three arms, we documented globally significant differences in all parameters examined by RCM, such as honeycomb pattern normalization and the absence of hyper/parakeratosis in the target lesion, together with an improvement in collagen rearrangement and solar elastosis in the superficial dermis of the FC.

### 2.2. Microscopic Features of Actinic Keratoses before and after Treatment

**Figure 2:**
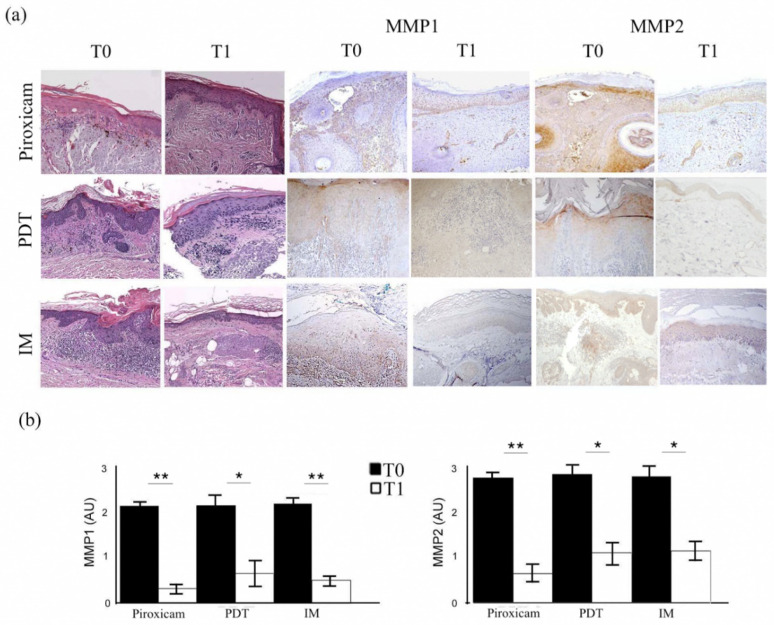
Histopathological features and metalloproteinase-1 and -2 expression in target lesions before (T0) and after treatment (T1). (**a**) The first two columns show the histopathological aspects of actinic keratoses before and after treatment with medical device 0.8% piroxicam and 50+ sunscreen, photodynamic therapy (PDT), and ingenol mebutate (IM) gel (hematoxylin–eosin, original magnification: 100×). The two central columns show images demonstrating the immunohistochemical expression of matrix metalloproteinase-1 (MMP1), while the last two show the staining for matrix metalloproteinase-2 (MMP2) in the target lesions before and after each treatment (original magnification: 100×). (**b**) Semiquantitative evaluation of matrix metalloproteinase expression before and after each therapeutic agent. * *p* < 0.01; ** *p* < 0.001.

The microscopic examination of the biopsies confirmed the absence of SCC features in all evaluated lesions and showed the typical morphological characteristics of AKs, such as keratinocytes dysplasia, spongiosis, dermal elastosis, and lymphocytic infiltrate (Figure 2a). According to the Rowert-Huber histopathological classification [1], there were 6 grade I AKs, 18 grade II AKs, and 6 grade III AKs. In each treatment group, there were two patients with grade I lesions, six patients with grade II lesions, and two patients with grade III lesions. After all treatments, the epithelial dysplasia disappearance as well as the improvement in dermal inflammatory infiltrate and elastosis were appreciated. Lesions treated with medical device piroxicam experienced an almost complete resolution of the lymphocytic infiltrate, while those cured with PDT showed a certain amount of chronic inflammation.

### 2.3. MMPs Evaluation before and after Treatments

**Figure 3:**
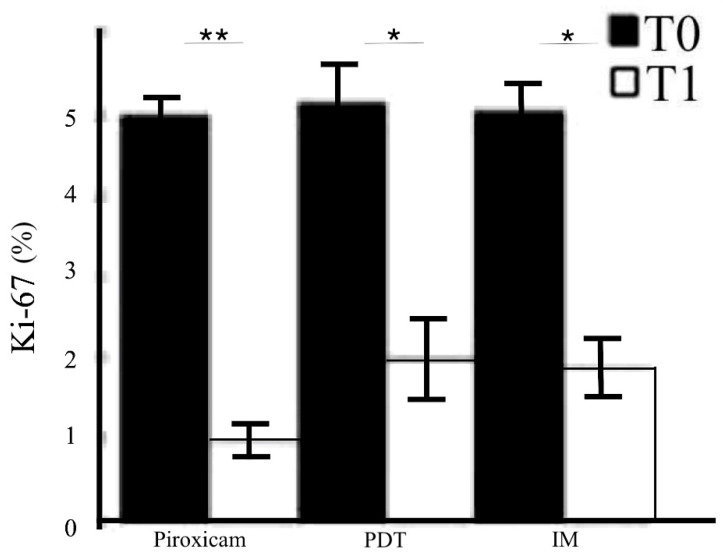
Immunohistochemical evaluation of the proliferation rate of the target lesions by Ki-67 before and after treatment. Semiquantitative evaluation of Ki-67 before and after medical device 0.8% piroxicam and 50+ sunscreen, photodynamic therapy (PDT), and ingenol mebutate (IM) gel. * *p* < 0.01; ** *p* < 0.001.

The immunohistochemical evaluation of MMP-1 and MMP-2 expression showed, in parallel with the clinical and histopathological features, significantly decreased staining in the treated skin compared to baseline (Figure 2a, right; *p* < 0.01). The MMP expression reduction was evident both in the dermis and in the epidermis. The reductions in MMP-1 and MMP-2 expression were similar when comparing the three treatments, as shown in Figure 2b, but were more notable for MMP-1 in group A (ANOVA *p* < 0.01). Ki67 epidermal immunopositivity also similarly declined after the end of the treatments (Figure 3; ANOVA *p* < 0.01), further supporting the efficacy of the three therapies of AKs.

### 2.4. Correlations between MMP Expression and Clinico-Pathological Features

Spearman’s analysis showed the following statistically significant correlations (*p* < 0.05): Pretreatment, a positive correlation between severe solar elastosis and both severe inflammation and a high histopathological grade of AK according to the Rowert-Huber classification (rho = 0.44 and 0.64, respectively) was observed. Furthermore, intense and diffuse MMP-1 and MMP-2 immunohistochemical expression correlated positively with both severe solar elastosis (rho = 0.41 and 0.52, respectively) and a high histopathological grade of AK (rho = 0.47 and 0.55, respectively) in most of the lesions evaluated in each treatment arm of the study.

Post-treatment, a positive correlation between decreased RCM score and both keratinocyte dysplasia resolution and solar elastosis improvement was established in AKs (rho = 0.47 and 0.61, respectively). Moreover, positive correlations were established between atypical honeycomb pattern normalization by RCM and a reduction in immunohistochemical MMP-1 and 2 expression in the target lesions (rho = 0.53 and 0.56, respectively). We noticed an improvement in collagen rearrangement observed in RCM and decreased MMP-1 and 2 in the FC (rho = 0.55 and 0.58, respectively). In particular, a positive correlation was observed between inflammatory infiltrate reduction, observed in RCM, and decreased MMP-1 expression (rho = 0.56) in group A. On the other hand, decreasing MMP-2 immunostaining expression was accompanied by strong inflammatory infiltrate, observed both by RCM and the histopathological examination, after PDT treatment (rho = 0.48).

## 3. Discussion

AKs and FCs with subclinical lesions are nowadays considered a chronic disease. They have to be treated early to avoid their evolution to SCC, which has higher costs in terms of morbidity and mortality. The currently available non-invasive techniques, used either for diagnostic or therapeutic monitoring purposes, are digital dermoscopy and RCM [20,30]. They both allow a suspect diagnosis that has to then be confirmed by histopathological examination, but the most promising results are obtained using RCM. It permits both in vivo and ex vivo diagnosis, evaluating the same lesion over time. Therefore, it is a useful tool not only to monitor the effectiveness of therapies but also to assess any recurrence in long-term follow-up [30]. The clinical, dermoscopic, and RCM results of this study confirm that the three compared treatments (medical device piroxicam, PDT, and IM) are effective in the management of AKs. The first aspect observed by RCM after the treatment was the gradual disappearance of hyper-parakeratosis, followed by the restoration of the honeycomb pattern in the target lesions, together with an improvement in the collagen rearrangement and solar elastosis in the superficial dermis of the FC. In both AKs and the FC, all treatments improved solar elastosis, an important sign of photodamage. Another peculiar aspect of our retrospective study was the evaluation of the immunohistochemical expression of two classes of MMPs both before and after each treatment. MMPs are involved in the degradation of different proteins of the ECM [31]. Keratinocytes and stromal cells secrete MMPs in response to oxidative stress, UV radiation, and inflammatory cytokines [32]. MMP-1 is a collagenase that is able to digest fibrillar collagen, while MMP-2 is a gelatinase that degrades collagen types I and IV [13]. Skin carcinogenesis is a multistep process beginning from intraepithelial malignant keratinocyte proliferation. It goes through an alteration in cell-to-cell adhesion and results in the degradation of the basement membrane and ECM, with the subsequent detachment and migration of tumor cells from the original location to distant sites [33,34]. The most effective barrier in preventing tumor invasiveness is represented by the basement membrane and the ECM. MMPs are implicated in the progression of AKs to SCC [12,33]. They play a central role in degrading these components. Thus, they are crucial therapeutic targets for the treatment and prevention of NMSCs [19]. The expression of MMP-1 is required in the initial steps of cutaneous tumor growth [13,33]. MMP-2 guarantees greater invasive properties, as demonstrated by comparing its expression in SCC and BCC [13,16,35]. The three different therapeutic modalities adopted in our study favored significant decreases in MMP-1 and 2 expression through their specific mechanisms of action. This result is associated with parallel decreases in the proliferation marker Ki-67 and, to a lesser extent, in the inflammatory infiltrate at the end of the three treatment modalities. In this regard, PDT induced a strong inflammatory infiltrate, likely a consequence of the release of ROS, which persists in the post-treatment period. Among the local adverse reactions, the medical device piroxicam caused only mild itching in 10% of patients, while IM showed erythema and skin desquamation in 30% patients. Photodynamic therapy only showed a burning sensation (20% patients) during the treatment session, redness after this, and in rare cases flaking (10% patients). All adverse effects were mild to moderate in intensity and did not cause the discontinuation of the treatments.

Our retrospective study has its roots in the absence of an established clinico-pathological correlation concerning the grading of dysplasia in AKs. Recently, the basal proliferation of dysplastic keratinocytes was proposed as a new histopathological criterion to explain AKs’ attitude to progress to SCC [36]. We investigated, from both clinical and histopathological points of view, the efficacy of three different therapeutic modalities on AKs and FCs, evaluating their biological effect and assessing the immunohistochemical expression of MMP-1 and 2. Above all, the strength of the study was exactly the evaluation of MMP-1 and 2 expression before and after the therapy, as those molecules are crucial in cutaneous epithelial carcinogenesis [13]. Considering the observed therapeutic effect and the healing of treated AKs, it is likely that the reduced expression of MMPs may be related to a local increase in tissue inhibitors of MMPs (TIMPs) or to the direct effect against inflammation mediated by the treatments.

The low percentage of piroxicam contained in the medical device adopted in this study has a synergistic action with the sunscreen, favoring the protection from UV damage of keratinocytes in chronic photodamaged skin [20,37,38,39,40]. It could therefore be considered a good option for treatment, as it acts on both the target lesions and on the FC and is extremely well tolerated. For patients with extensive skin photodamage at risk for multiple NMSCs, it could be considered a sort of tailored therapy, mainly when they are treated with photosensitizing drugs, such as antihypertensives [27], antibiotics, and chemotherapy [41]. Moreover, it could be used in combination with a session of PDT if the AKs are extensive and severe.

In conclusion, the value of this retrospective study is the correlation of clinical and histopathological findings after three different therapeutic approaches to treat AKs and FCs, which have to be considered chronic diseases involving a growing portion of the population, including young patients. The three treatment modalities play a role in the different pathogenetic phases of the disease and affect specific targets. They could be used in a combined and/or sequential regimen, depending on the period of the year. The pathogenetic role of MMPs in the development and evolution of AK is largely known, and dermatologists should consider therapies with renowned effectiveness on these targets to prevent the progression to SCC. In this study, it was possible to evaluate the high efficacy of the treatments and consider the low incidence and mild intensity of the adverse reactions, confirming the safety of and tolerance to these therapies, especially piroxicam, which is also a candidate for AK treatment in immunosuppressed patients (as it does not stimulate immunogenicity in transplant recipients). Considering the high prevalence of AKs in the general population, not only due to irresponsible UV exposure but also to treatment with photosensitizing drugs and environmental pollutants (as a consequence of climate change), the chance to treat these lesions permits the control of costs in all those patients who would have developed SCC. Further studies may be encouraged to confirm our results in larger cohorts also considering the risk factors to understand which would be the best treatment choice in each group. Finally, even though it presented a satisfactory action in reducing MMP expression, the license of IM has recently been suspended by EMA for some pharmacovigilance concerns. It would be interesting to evaluate whether these good results could also be confirmed using other immunomodulatory substances, such as imiquimod.

## 4. Methods and Patients

### 4.1. Patients

The present retrospective study included 30 patients, 24 men and 6 women with a mean age of 74 years (range 60–88 years; Table 1) and a previous history of multiple NMSCs. The study protocol consisted of three groups of patients selected in the database of the Dermatologic Unit of Tor Vergata University Hospital of Rome, Italy, from July 2017 to December 2018 that were homogeneous for both patient and lesion features (Table 1). The first group of patients (group A, *n* = 10) was treated with medical device piroxicam twice a day for 16 weeks. The second group (group B, *n* = 10) was treated with two sessions of PDT using methyl aminolevulinate. The third group (group C, *n* = 10) was treated with a single cycle of 0.015% or 0.05% IM (which was still authorized in the period under consideration in the study) once daily for three days for lesions on face and scalp or trunk and extremities, respectively. An area ≥25 cm² was treated for each patient, including both the FC and the target lesions. The study was conducted in accordance with the Declaration of Helsinki and approved by the local institutional ethical committee (n. 216-17). The patients included in the study were older than 18, signed an informed written consent, and had at least three lesions in an area ≥25 cm² of the FC. Moreover, only patients who had tissue samples of the target lesions were selected for the study. Cutaneous specimens were collected by performing 3 mm punch biopsies at the sites of target lesions strongly suspicious for SCC before treatment and at the end of the therapy to exclude residual keratinocytic dysplasia. We then investigated the histopathological features and quantified the immunohistochemical markers [42]. The clinical classification of the AKs was established according to the evidence- and consensus-based (S3) guidelines of the International League of Dermatological Societies [25]. The exclusion criteria were metastatic neoplasms, invasive melanoma or another malignancy in the past 5 years, and topical therapies or FC treatments for AKs in the previous 8 weeks.

### 4.2. Dermoscopic and RCM Evaluation

The diameters of target lesions ranged from 5 to 15 mm. In the treatment area, we identified the clinically evident target lesions on an FC. The clinical features, dermoscopy, and RCM assessments were collected for every lesion of each patient for all three arms of the study at baseline, during therapy, and at the endpoint. The dermoscopy of AK is characterized by the presence of specific diagnostic criteria, and the three different clinical grades of AKs, according to Olsen, corresponded dermoscopically to three different models (Table 2) [43]. Only grade 1 and 2 AKs, according to Olsen’s classification, were included in the study. For the score in RCM, we took into consideration different characteristics of the corneum, granular, and spinous layers and the dermis [44]. Table 3 and Table 4 show the parameters used for the definition of a score for the dermoscopic and RCM evaluations before and after treatment. Dermoscopy was used only on target lesions, while the RCM examination was performed both on the target and on the FC.

### 4.3. Histopathological and Immunohistochemical Evaluation

The morphology of the biopsied lesions was evaluated by serial sections stained with hematoxylin–eosin. For the immunohistochemical analysis, sections of 4–5 μm thickness were cut at the microtome and placed on positively charged slides [20]. After deparaffination, in order to block the endogenous peroxidases, the sections were treated with 0.2% H_2_O_2_ in methanol for 20 min at room temperature and subsequently subjected to retrieval for 30 min at 98 °C in citrate buffer (10 mM sodium citrate, pH 6.0) or in EDTA-citrate buffer as required by the data sheets of the different antibodies. The sections were then incubated with anti-MMP-1 and MMP-2 (Thermo Fisher Scientific, Waltham, MA, USA) and with Ki-67 antibodies (Dako, Los Angeles, CA, USA). After this, the sections were incubated with antibodies against rabbit or mouse IgG and streptavidin ABC (Dako, Los Angeles, CA, USA) using 3,3-diaminobenzidine (DAB) as a chromogen. The evaluation of sections stained by immunohistochemistry was performed by two independent researchers with an interobserver variability <5%.

### 4.4. Statistical Analysis

For quantitative variables, data were analyzed by an ANOVA or Student’s *t*-test. The univariate analysis of the relationship between the clinico-pathological variables and the markers was performed using Spearman’s rank test. The differences were considered statistically significant for *p*-values < 0.01. The statistical analysis was performed with SSPS V20 (Stat Corp, College Station, TX, USA).

## Figures and Tables

**Table 1:** Demographic and clinico-pathological data of enrolled patients before treatments (*n* = 30). Abbreviations: AK, actinic keratosis.

Variable		N	Group A (Medical Device 0.8% Piroxicam + 10+ Sunscreen) (*n* = 10)	Group B (Photodynamic Therapy) (*n* = 10)	Group C (Ingenol Mebutate Gel) (*n* = 10)
Age (mean = 74.5; range 60–88)	>74.5	11	74.8	74.0	74.2
	<74.5	19			
Sex	Male	24	8	8	8
	Female	6	2	2	2
Anatomic location	Head/neck	21	7	7	7
	Extremities	9	3	3	3
AK clinical grade (according to Olsen)	1	13	4	4	5
	2	17	6	6	5
	3	0	0	0	0
AK histopathologic grade (according to Rowert-Huber)	I	6	2	2	2
	II	18	6	6	6
	III	6	2	2	2
Solar elastosis	Mild	5	2	1	2
	Moderate	11	3	4	4
	Severe	13	5	4	4
Peritumoral inflammatory infiltrate	Mild	8	3	3	2
	Moderate	13	4	4	5
	Severe	9	3	3	3

**Table 2:** Comparison between clinical and corresponding dermoscopic features of actinic keratoses according to grading.

Grade	Clinical Classification (Olsen’s)	Dermoscopic Classification [43]
1	Skin-colored macule without hyperkeratosis (easier felt than seen)	- red pseudonetwork pattern; - white scales
2	Moderate hyperkeratotic lesion in an erythematous background (easily felt and seen)	- white to yellow, enlarged, keratotic follicular openings in an erythematous background (“strawberry pattern”)
3	Very thick and hyperkeratotic lesion, which may include SCC in the differential diagnoses	- enlarged and keratin-filled follicles over a scaly background; - structureless areas with marked hyperkeratosis

**Table 3:** Dermoscopic parameters used for the definition of a score for the target lesion of each patient before and after treatment.

**Erythema**	0 = absent from 1 to 4 = present (4 = maximum erythema)
**Scaling**	0 = absent from 1 to 4 = present (4 = maximum scaling)
**Pigmentation**	0 = absent 1 = light and focal pigmentation from 2 to 4 = increasing level of brownish pigmentation 5 = dark pigmentation
**Follicular plugs**	0 = absent 1 = few plugs in just one of the evaluated areas 2 and 3 = increasing number of plugs in several areas 4 = diffuse presence of follicular plugs

**Table 4:** Reflectance confocal microscopy parameters used for the definition of a score for the target lesion and the field of cancerization of each patient before and after treatment.

Corneum Layer	Absence	Presence
Scale	0	1
Dysruption/detached corneocytes	0	1
Parakeratosis	0	1
Polygonal keratinocytes	0	1
**Granular and spinous layers**		
Atypical honeycomb	0	1
Inflammatory cells	0	1
Round nucleated cells	0	1
**Dermis**		
Curled fibres	0	1
Collagen alterations	0	1
Increased vascularity	0	1
Inflammatory cells	0	1
Melanophages	0	1

## Data Availability

Data are available from the corresponding author on reasonable request.
